# Cyclotides from Plants Driving the Next Generation of Antibacterial Agents

**DOI:** 10.3390/antibiotics15060604

**Published:** 2026-06-13

**Authors:** Elizabete de Souza Cândido, Liryel Silva Gasparetto, Mariana Rocha Maximiano, Thuanny Borba Rios, Octávio Luiz Franco

**Affiliations:** 1 Programa de Pós-Graduação em Ciências Genômicas e Biotecnologia, Universidade Católica de Brasília, Brasilia 71966-700, Brazil; betty.souza@gmail.com (E.d.S.C.); liryelg@gmail.com (L.S.G.); marianamaximianor@gmail.com (M.R.M.); thuannyborba@gmail.com (T.B.R.); 2 S-Inova Biotech, Programa de Pós-Graduação em Biotecnologia, Universidade Católica Dom Bosco, Campo Grande 79117-900, Brazil

**Keywords:** cyclotides, antibacterial, plant, peptides, cyclic peptides, bacteria

## Abstract

**Background/Objectives**: Cyclotides are plant-derived macrocyclic peptides distinguished by their head-to-tail cyclized backbone and cystine knot motif, which confer remarkable stability against thermal, enzymatic, and chemical degradation. These features, combined with a compact and rigid structure, position cyclotides as promising scaffolds for future antibacterial agents in response to the escalating threat of multidrug-resistant (MDR) pathogens and the stagnation of conventional antibiotic discovery pipelines. This review summarizes the structural features, antibacterial mechanisms, bioengineering strategies, and translational potential of cyclotides against MDR infections. **Methods**: A narrative review of the literature was conducted using recent original research articles and reviews on cyclotide structure, antibacterial activity, bioengineering, computational modeling, and pharmaceutical applications. **Results**: Cyclotides exhibit potent antimicrobial activity, primarily through membrane disruption mediated by amphipathic surfaces and affinity for anionic bacterial membranes. Some variants also demonstrate anti-virulence and antibiofilm properties, broadening their therapeutic relevance for difficult-to-treat infections. Bioengineering approaches, including epitope grafting and rational design, have improved selectivity and potency while reducing cytotoxicity. Advances in computational modeling, molecular dynamics, and artificial intelligence have accelerated the prediction and optimization of antimicrobial activity, toxicity, and pharmacokinetic properties. **Conclusions**: Innovations in synthesis, including recombinant expression and enzymatic ligation, are helping overcome translational barriers related to cost and scalability. Although challenges remain in oral bioavailability and systemic delivery, strategies such as lipidation and scaffold modification support the development of cyclotide-based therapeutics as adaptable platforms for peptide drug discovery.

## 1. Introduction

With the global rise of antimicrobial resistance, developing alternative strategies for the effective treatment of bacterial infections has become a critical priority [1]. Conventional antibiotics are losing efficacy against multidrug-resistant (MDR) bacteria, and few truly novel antibacterial agents are advancing through clinical trials, leaving a pressing gap in our therapeutic arsenal [1,2]. In this context, antimicrobial peptides (AMPs) represent promising candidates due to their broad-spectrum activity and ability to target diverse pathogens [3,4]. These plant-derived AMPs can be categorized into multiple families characterized by distinct structural motifs and biological functions, including cyclotides, defensins, thionins, and hevein-like peptides, each offering unique opportunities for therapeutic development [5].

Among these, cyclotides have emerged as a particularly compelling alternative. These small peptides (~30 amino acids) feature a unique head-to-tail cyclic backbone stabilized by a knotted arrangement of six cysteine residues that form three disulfide bonds, collectively known as the cystine cyclic knot (CCK) motif [6,7]. Their compact size facilitates chemical synthesis, recombinant production, and tissue penetration compared to many larger AMPs, whose size can limit bioavailability and increase production cost. Their distinctive topology sets them apart from conventional linear AMPs and cyclic peptides, imparting exceptional resistance to proteolytic degradation and to thermal and chemical denaturation, thereby making them attractive candidates for systemic administration, oral delivery, and long-term storage [8].

Initially discovered in members of the Violaceae family, cyclotides exhibit broad-spectrum antimicrobial activity, including activity against notoriously resistant Gram-negative pathogens and biofilm-forming bacteria [7,8]. Their potent activity against these clinically relevant pathogens positions cyclotides as promising candidates to address infections that currently evade antibiotics. Interestingly, cyclotides also exist in a linear form, known as acyclotides. Despite lacking the characteristic head-to-tail cyclization, acyclotides maintain the conserved cystine knot structure formed by three disulfide bonds [9]. This conserved motif ensures that acyclotides maintain a stability level comparable to that of their cyclic counterparts, thereby broadening the potential structural and functional diversity of this peptide family [8,9].

Cyclotides have been isolated from eight plant families, including Violaceae, Rubiaceae, Solanaceae, Cucurbitaceae, Poaceae, Apocynaceae, Euphorbiaceae, and Fabaceae [10,11]. Generally, they are grouped into three main subfamilies, differentiated primarily by the presence or absence of a cis-proline residue within loop 5. The Möbius subfamily is characterized by a backbone twist induced by this cis-proline, whereas the bracelet subfamily lacks the proline and exhibits a more relaxed conformation. A third group consists of trypsin inhibitors, which show divergent primary sequences yet still retain the conserved cystine knot architecture; all retain the hallmark cystine knot that underpins their remarkable stability and sequence tolerance [9]. Acyclotides fit within these same classifications [9]. The cystine knot motif enables substantial sequence variability without compromising structural integrity [8].

Despite their exciting antimicrobial properties, translating cyclotides from natural defense molecules into clinically viable antibacterial therapies remains a largely conceptual effort. Key translational barriers include potential cytotoxicity to human cells, immunogenicity, challenges in scalable synthesis, and limited pharmacokinetic characterization data [7,8]. Yet, ongoing innovations such as enzymatic cyclization methods, computational redesign of bioactive loops, and conjugation to delivery platforms are actively addressing these barriers, paving the way for clinical application [8,12].

Despite these advances, several limitations persist. Cyclotides can exhibit variable hemolytic and cytotoxic activity, often influenced by sequence and net charge [7,11]. Moreover, while their stability supports pharmaceutical development, challenges related to immunogenicity, delivery, and large-scale synthesis remain to be fully addressed [8]. Nonetheless, these concerns are being actively investigated through rational design, scaffold engineering, and new biotechnological synthesis methods. In addition, the inherent modularity of the cyclotide scaffold allows for strategic sequence variation to enhance antibacterial potency while minimizing adverse effects, underscoring its versatility as a therapeutic platform [8,12].

Previous reviews have broadly discussed cyclotides as therapeutic scaffolds, but few have specifically focused on their antibacterial potential and translational development. In addition, recent advances in peptide engineering, chemical synthesis, and artificial intelligence-assisted design for antimicrobial drug development have not been comprehensively integrated. This review addresses these gaps by focusing on the discovery, antibacterial mechanisms, engineering strategies, and translational challenges of cyclotides against multidrug-resistant pathogens.

This narrative review summarizes current knowledge on cyclotides as antibacterial scaffolds and their translational potential. Literature searches were conducted in PubMed, Scopus, Web of Science, and Google Scholar using keywords related to cyclotides, cyclic peptides, antimicrobial activity, multidrug resistance, biofilms, anti-virulence strategies, peptide engineering, chemical synthesis, AI, drug development, and pharmacokinetics. Priority was given to peer-reviewed articles published between 1990 and January 2026 that reported experimentally validated antibacterial activity, structure–activity relationships, engineering strategies, or translational applications. Foundational studies outside this timeframe were included when relevant, while studies focused solely on non-antibacterial applications without translational relevance were excluded.

## 2. Structure–Activity Relationships Underlying the Antibacterial Functions of Cyclotides

**Figure 1:**
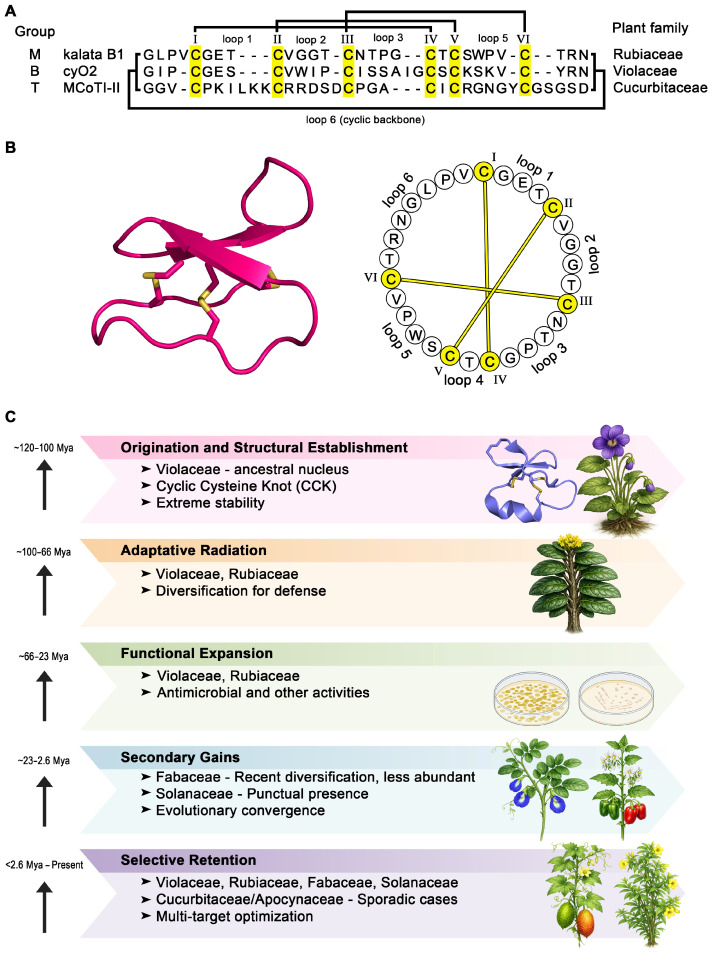
(**A**) Sequences of kalata B1 (Rubiaceae), cycloviolacin O2 (Violaceae), and MCoTI-II (Cucurbitaceae), representative members of the Möbius (M), bracelet (B), and trypsin inhibitor (T) cyclotide subfamilies, respectively. The sequences highlight the six conserved cysteine residues (Cys I–VI) in yellow and the six inter-cysteine loops (loops 1–6) in black, exhibiting the distinct sequence features and loop lengths of each subgroup. (**B**) Conserved cyclotide 3D structure featuring a head-to-tail cyclized backbone and the CCK motif. The six conserved cysteine residues form three disulfide bonds (in yellow) with a characteristic I–IV, II–V, and III–VI connectivity. The three-dimensional structure of kalata B1 was generated from the experimental structure deposited in the Protein Data Bank (PDB ID: 1NB1) and rendered using PyMOL version 3.1. (**C**) Evolutionary framework of plant cyclotides—proposed timeline summarizing the origin, diversification, and retention of cyclotides in angiosperms. Evolutionary representation is intended as a conceptual illustration based on currently available phylogenetic evidence and not as a reconstructed evolutionary timeline. Cyclotides likely originated in Violaceae during the late Cretaceous with the establishment of the CCK motif. Subsequent adaptive radiation in Violaceae promoted sequence diversification associated with plant defense. Functional expansion includes the emergence of antimicrobial and related bioactivities. More recent secondary gains in Fabaceae and sporadically in Solanaceae suggest convergent evolution. From the Pleistocene to the present, cyclotides have been selectively retained across multiple plant families.

Although all cyclotides share the conserved CCK motif, they show structural and functional diversity across plant families, reflecting evolutionary adaptation to distinct ecological pressures. Variations in loop length, amino acid composition, net charge, and hydrophobic surface distribution give rise to a broad spectrum of biological activities [13]. Cyclotides from the Violaceae and Rubiaceae families, for example, frequently display strong membrane-active antimicrobial properties. In contrast, those from Cucurbitaceae and Fabaceae are often optimized as protease inhibitors involved in herbivore defense. In particular, the trypsin inhibitor cyclotide sequence and loop architecture differ markedly from those of the Möbius and Bracelet subgroups (Figure 1A) and show homology to knottins, a family of typically linear peptides [13]. Notably, this diversity remains incompletely explored: cyclotides are frequently present in the Violaceae family, whereas they are comparatively sparse in other families, occurring in fewer than 5% of screened Rubiaceae species and only sporadically in several additional plant lineages. Moreover, individual plant species typically produce a unique repertoire of cyclotides, with little overlap among species [13].

At the structural level, cyclotides are defined by a head-to-tail cyclized peptide backbone stabilized by three disulfide bonds arranged in a cystine knot topology (Figure 1B). This architecture enforces a compact, rigid three-dimensional fold that confers stability and functional versatility [7]. The constrained backbone presents amphipathic surfaces with precisely positioned hydrophobic and charged residues, enabling efficient interactions with biological membranes and other molecular targets. Notably, the CCK motif imparts properties rarely achieved concurrently in linear antimicrobial peptides, such as extraordinary resistance to proteolytic degradation, pH extremes, and thermal stress [14]. Despite this rigidity, cyclotides tolerate extensive sequence variation within solvent-exposed loops, allowing fine-tuning of physicochemical properties and biological function without compromising structural integrity [9]. This unique combination of stability and mutational tolerance reinforces the appeal of cyclotides as robust scaffolds for peptide engineering.

Beyond their antibacterial effects, cyclotides exhibit a broad range of bioactivities that further enhance their therapeutic and biotechnological relevance, with several reported to display insecticidal, nematicidal, and anti-HIV activities, among other activities [15,16,17]. Their stability enables sustained bioactivity in complex biological environments, while their compact size facilitates tissue penetration and molecular target engagement. Importantly, the modular architecture of cyclotides allows specific bioactive epitopes to be embedded within a stable framework, supporting multifunctional activity profiles that extend well beyond membrane disruption [7,11]. These diverse bioactivities emphasize that cyclotides should be viewed not merely as AMPs, but as versatile molecular platforms with broad biomedical potential.

As with many membrane-active peptides, host toxicity remains an important consideration in cyclotide development. Certain cyclotides can exhibit hemolytic or cytotoxic effects toward mammalian cells. They bind preferentially to membranes containing phosphatidylethanolamine (PE), insert via a hydrophobic surface patch, and then permeabilize or disrupt the bilayer by surface-exposed hydrophobic residues and elevated net positive charge [8,18]. Crucially, these effects are highly sequence-dependent and not intrinsic to the cyclotide scaffold itself. Structure–activity relationship studies have shown that targeted modification of surface residues can significantly reduce hemolysis while preserving or even enhancing antibacterial activity, underscoring the tunability of the scaffold [18].

Data on cyclotide immunogenicity in vivo remain limited, and reviews highlight potential immunogenicity as an outstanding hurdle for therapeutic development [16]. However, available experimental evidence suggests that some native cyclotides and engineered analogs are weakly immunogenic or even immunologically silent, particularly when used as inert scaffolds [19]. Cyclotides can become immunogenic when deliberately targeted to antigen-presenting cells, underscoring that immune activation is highly context- and design-dependent rather than an intrinsic property of the scaffold itself [19]. Together with reported concerns regarding cytotoxicity and hemolysis, these observations emphasize the need for systematic in vivo evaluation of immunogenicity alongside toxicity profiling during the development of cyclotides as therapeutic agents.

From a production perspective, cyclotides were initially discovered through extraction and purification from plant material, a strategy that remains valuable for uncovering natural diversity but is limited by low yields, seasonal variability, and poor scalability. Notably, production varies across cyclotide subfamilies. Bracelet cyclotides, despite constituting approximately two-thirds of all known cyclotides, are generally larger and structurally more complex than Möbius or trypsin inhibitor cyclotides. As a result, they are more challenging to fold in vitro, which limits their use as engineering scaffolds and makes Möbius and trypsin inhibitor cyclotides the preferred frameworks for synthetic modification and therapeutic development [14,20].

Figure 1C illustrates how cyclotides represent a notable example of evolutionary optimization within the plant kingdom, combining exceptional structural stability with distinct functional diversity [6,13]. It is evident that a highly conserved molecular scaffold, as already discussed here, the CCK, is preserved across distinct plant families, while sequence variability is selectively concentrated in surface-exposed loop regions. This dual strategy reconciles two opposing evolutionary demands, including long-term structural stability and rapid functional adaptation driven by biotic pressures. The widespread conservation of the CCK motif across phylogenetically distant plant families, including Violaceae, Rubiaceae, Fabaceae, and, more infrequently, Solanaceae, supports the hypothesis that cyclotides originated early in angiosperm evolution and were subsequently maintained under strong positive selection [21,22]. The cyclic backbone, combined with linked disulfide bonds, confers resistance to proteolytic degradation, thermal denaturation, and chemical stress, properties central to their ecological function as plant defense peptides [13].

In divergence from the conserved structural core, the loops linking the cysteine residues exhibit marked hypervariability in both length and amino acid composition. This pattern is consistent with adaptive evolution driven by coevolutionary interactions with herbivores, insects, and microbial pathogens [7,15]. Modulation of charge, hydrophobicity, and amphipathic surfaces within these loops directly influences membrane affinity and target selectivity, accounting for the wide range of biological activities described for cyclotides, including antimicrobial, insecticidal, cytotoxic, and antiviral effects [23,24]. This evolutionary approach supports the conserved scaffold–variable surface standard observed in other antimicrobial peptide families, reinforcing cyclotides’ position as privileged molecular frameworks. Family-specific diversification patterns further indicate that cyclotide evolution is shaped not only by phylogeny but also by ecological context [21]. The Violaceae family is widely recognized as a major reservoir of cyclotide diversity, exhibiting extensive gene duplication and sequence diversification, consistent with long-term evolutionary retention [25]. In contrast, the sporadic distribution of cyclotides in families such as Fabaceae and Solanaceae may reflect secondary acquisition events, lineage-specific losses, or ecological specialization, followed by functional refinement under localized selective pressures [26].

From a translational standpoint, the evolutionary success of cyclotides provides valuable insights for peptide engineering and therapeutic development. Their exceptional stability, combined with an intrinsic tolerance for sequence variation in functional loops, supports their application as scaffolds for drug design and molecular grafting [13]. Understanding sequence variation, based on evolutionary principles, can guide rational peptide design strategies, refining bioactivity and bioavailability while preserving structural integrity and pharmacokinetic stability. In this scenario, cyclotides exemplify how natural evolutionary solutions can be leveraged for modern peptide-based technologies. In summary, the evolutionary trajectory of cyclotides could be defined by an initial structural fixation followed by extensive functional diversification driven by ecological interactions. This balance between conservation and innovation may explain both their persistence in multiple plant families and their growing relevance in peptide science.

## 3. Mechanisms, Spectrum, and Potency of Antimicrobial Cyclotides

Cyclotides are increasingly recognized for their potent and broad-spectrum antimicrobial activity. The biological basis for this activity lies primarily in their ability to disrupt bacterial membranes, driven by their amphipathic character and constrained cyclic backbone, which present hydrophobic and positively charged residues in precise spatial arrangements optimized for membrane interaction [6,8]. These structural characteristics enable a strong electrostatic attraction to the negatively charged outer membranes of bacteria, followed by the insertion of hydrophobic patches that destabilize lipid bilayers and ultimately lead to rapid membrane permeabilization [8,13,27].

Recent molecular dynamics simulations have deepened mechanistic understanding, revealing that the CCK motif not only imparts exceptional proteolytic, thermal, and chemical stability but also locks cyclotides into bioactive conformations critical for membrane insertion [8]. Complementary in silico studies have proposed that some cyclotides might also inhibit bacterial virulence factors, suggesting dual mechanisms that extend beyond purely lytic action [12].

The antibacterial spectrum of cyclotides is particularly notable. Studies have reported activity against a range of clinically relevant Gram-negative bacteria, including *Escherichia coli*, *Pseudomonas aeruginosa*, and *Acinetobacter baumannii*, as well as Gram-positive species such as *Staphylococcus aureus* [8,13,28]. The high activity toward Gram-negative bacteria is often linked to higher densities of anionic phospholipids in their outer membranes [28].

Natural cyclotides, such as cycloviolacin O2 (cyO2), have been reported to exhibit low-micromolar MICs (Minimum inhibitory concentrations) against multiple Gram-negative bacteria. In contrast, cationic cyclotides, such as cT15 and cT19, selectively target Gram-negative bacteria by exploiting their more anionic outer membranes [13,29]. Importantly, engineering efforts have also produced broad-spectrum candidates. For example, MCo-PG2, created by grafting the protegrin-1 loop onto a cyclotide scaffold, achieved MIC_50_ values as low as 1.5 µM against MDR *P. aeruginosa* and rescued over 90% of mice in a peritonitis model, outperforming colistin [30].

While only a limited subset of natural cyclotides has been extensively studied for antibacterial effects, such as kalata B1/B2, circulin A/B, and cycloviolacin O2, continued screening of plant extracts promises to expand this repertoire. For instance, a recent study identified the novel cyclotide gere1 from *Geophila repens*, which exhibited an MIC of 4 µM against *E. coli*, highlighting the untapped potential of cyclotides [31]. Despite the lack of a definitive list of naturally expressed cyclotides with antibacterial activity, Table 1 presents a concise collection of cyclotides reported to date that act as antibacterial agents, primarily targeting bacterial membranes.

Beyond planktonic bacteria, some authors have reported antibiofilm effects under experimental conditions, a crucial property given the role of biofilms in chronic and device-associated infections. Although the precise mechanisms underlying cyclotide antibiofilm activity are not yet fully elucidated, it is believed that cyclotides disrupt membranes and potentially interfere with adherence, thereby reducing biofilm biomass and viability [40]. In addition to direct bactericidal activity, cyclotides may interfere with early stages of biofilm development, such as bacterial adhesion and surface colonization, ultimately reducing biofilm biomass and viability [40]. However, mechanistic evidence supporting more specific actions, such as interference with quorum-sensing pathways, modulation of biofilm-related gene expression, or degradation of extracellular matrix components, remains scarce and requires further investigation.

Early studies have begun to explore cyclotide-based coatings as preventive strategies, utilizing cyclotide fractions from *Viola philippica*. The modified surfaces showed a significant reduction in S. aureus adhesion and biofilm biomass compared with untreated stainless-steel controls. Scanning electron microscopy analyses further revealed fewer attached bacterial cells and noticeable alterations in cellular morphology on cyclotide-coated surfaces, supporting the hypothesis that the antibiofilm effect is primarily attributable to localized antibacterial activity at the material interface [40]. These findings highlight the potential of cyclotides as antibiofilm agents for biomedical devices and implantable materials. Still, this is undoubtedly a field to be explored, given the scarcity of data that permeates this possibility. Their exceptional stability against thermal, chemical, and enzymatic degradation further strengthens their suitability for surface-functionalization strategies in clinical settings [13,40]. Nevertheless, the available literature remains limited, and substantial gaps persist regarding their spectrum of antibiofilm activity, efficacy against mature biofilms, long-term surface stability, and possible synergistic interactions with conventional antibiotics.

One challenge for clinical translation remains host toxicity, notably hemolytic effects towards human erythrocytes. Because their activity operates via membrane disruption, a lack of selectivity can lead to toxicity toward mammalian cells, particularly red blood cells. Cyclotides show moderate hemolytic potential compared with melittin [27,41,42].

Several naturally occurring cyclotides exhibit antibacterial activity at concentrations near those that induce hemolysis, thereby narrowing their therapeutic window. For example, the bracelet cyclotide cyO2 exhibits potent bactericidal activity against Gram-negative bacteria with MIC values against *E. coli* (~25 µM, MIC_50_~6.8 µM) being reported in the same range as its hemolytic IC_50_ (~5 µM), reflecting the partial overlap between bacterial membrane disruption and erythrocyte membrane destabilization and indicating poor selectivity between bacteria and erythrocytes [27,28,42]. Similarly, studies evaluating kalata B1 and related cyclotides demonstrated that phosphatidylethanolamine-binding properties contribute to antibacterial activity but may also influence interactions with mammalian membranes depending on peptide hydrophobicity and charge distribution [8,42].

Importantly, not all cyclotides exhibit the same toxicity profile. Differences in loop composition, surface hydrophobicity, net charge, and amphipathic organization strongly influence membrane selectivity and can substantially alter the balance between antibacterial potency and hemolytic activity [43]. Consequently, comparison between MIC values and hemolytic concentrations is essential when evaluating the therapeutic potential of engineered cyclotides, as antibacterial activity alone may overestimate translational applicability.

Interestingly, studies have demonstrated that the hemolytic activity is lost upon cyclotide linearization, indicating the need for an intact peptide structure [27,42]. Hemolysis tends to correlate with a net positive charge and the distribution of hydrophobic residues [13]. Still, it is suggested that exposure of hydrophobic amino acid residues on the peptide surface favors interactions with, and disruption of, lipid bilayers [42]. Encouragingly, structure–activity studies have guided rational design, showing that modifying surface residues can reduce hemolytic activity while preserving antimicrobial potency [8,42]. This fine-tuning is enabled by the scaffold’s structural rigidity, which supports targeted mutations without destabilizing the fold [43].

Overall, cyclotides combine broad-spectrum antibacterial activity, exceptional stability, design flexibility, and emerging antibiofilm and anti-virulence effects. These features distinguish them from many conventional antibiotics and position them as compelling candidates in the development of next-generation peptide therapeutics against multidrug-resistant infections [44].

## 4. Translational Barriers and Bioengineering Strategies

Recent years have witnessed a convergence of bioinformatics, molecular modeling, and rational design that is transforming the optimization of cyclotides for antibacterial therapy. These tools capitalize on their unique properties, allowing for enhanced potency, spectrum, and safety, which characterize key steps toward clinical translation.

In silico methods have emerged as powerful tools for screening cyclotides as antibacterial agents, allowing researchers to bypass labor-intensive experimental screens and rapidly assess therapeutic potential. For example, protein-peptide docking enables the virtual screening of cyclotide libraries against bacterial targets, facilitating the identification of molecules with novel mechanisms of action [12]. Recent studies have predicted, via protein-peptide docking, the binding of cyclotide candidates to neuraminidase, a key virulence factor in Streptococcus pneumoniae, revealing opportunities to inhibit bacterial pathogenicity rather than directly killing cells [12]. This example illustrates how computational approaches can expand the antibacterial potential of cyclotides beyond membrane disruption, uncovering candidates that modulate virulence pathways and complement conventional antibiotic strategies.

**Figure 2:**
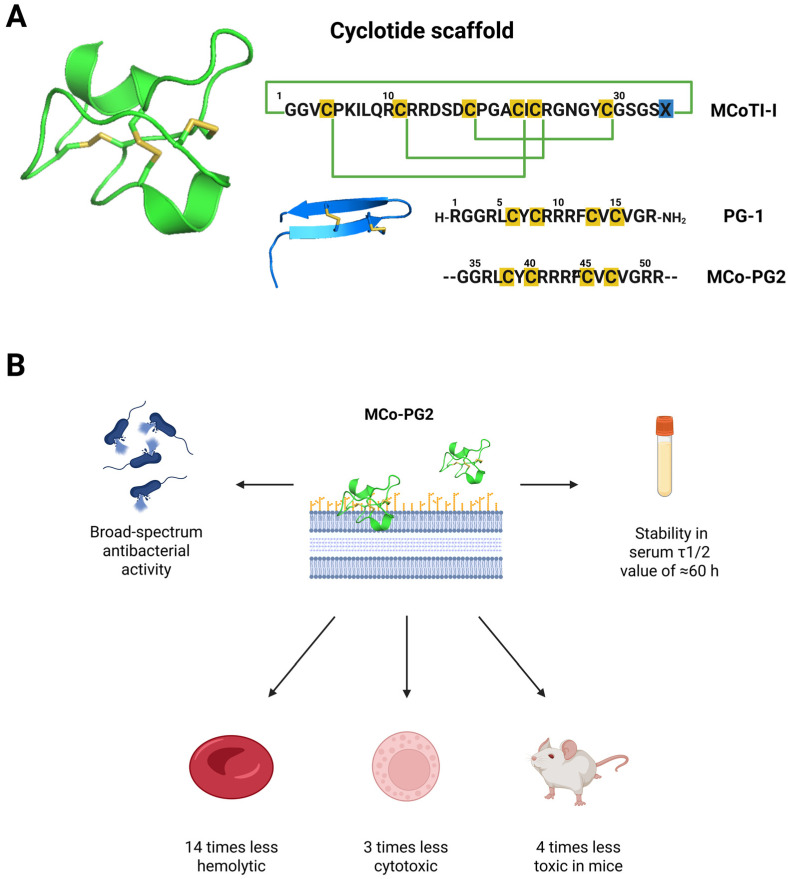
Enhancement of activity through epitope grafting on a cyclotide scaffold. (**A**) Schematic representation of the strategy used to design MCo-PG2. A circularly permuted version of porcine protegrin PG-1, in which the original Arg^1^ residue was relocated to the C-terminus, was grafted into loop 6 of the cyclotide MCoTI-I, between Gly^1^ and Ser^33^. The resulting backbone-cyclized structure is shown with the cyclization bond highlighted in green. Cysteine residues are shown in yellow, and the three disulfide bonds are shown as green connecting lines. Ribbon diagrams of the parental cyclotide MCoTI-I (PDB: 5WOV) and porcine protegrin PG-1 (PDB: 1PG1) are shown for reference. (**B**) MCo-PG2 exhibited broader antimicrobial activity compared to the parental cyclotide, as well as increased serum stability, reduced hemolysis, and lower cytotoxicity both in vitro and in vivo (mice) when compared to the parental PG-1.

Likewise, molecular dynamics simulations showed how the CCK knot motif locks cyclotides into conformations that preserve membrane-active amphipathic surfaces, explaining both their stability and efficacy [8]. These computational approaches complement traditional screening, allowing rapid prediction of activity, toxicity, hemolysis, and even stability [8,12,45]. At the design level, the defining feature of cyclotides, the CCK motif, confers not only thermal and proteolytic stability but also remarkable tolerance for sequence variability and precise mutations [7,30]. This property makes cyclotides especially attractive scaffolds for rational engineering against bacterial infections, where stability and adaptability are critical. One powerful strategy is epitope grafting, which involves inserting short bioactive antimicrobial peptide sequences into the loops of a cyclotide backbone, thereby creating chimeric molecules that combine the scaffold’s stability with the grafted motif’s antimicrobial function [30]. For instance, the engineered chimera MCo-PG2, which incorporates the protegrin-1 loop into the MCoTI-I scaffold (Figure 2A), achieved MIC_50_ values as low as 1.5 µM against MDR *P. aeruginosa* and 6.25 µM against MDR *S. aureus*, showing a successful insertion of new functional motifs while maintaining the overall fold and stability (Figure 2B) [30].

While loops 1 and 4 are structurally constrained by the disulfide knot, loops 2, 3, 5, and 6 can accommodate diverse grafts [44]. This adaptability has been exploited to insert antimicrobial epitopes targeting specific pathogens, biofilms, or bacterial virulence factors. Importantly, grafted cyclotides preserve the native fold and can enhance proteolytic resistance and bioavailability compared to linear peptides [22].

In silico bioinformatics approaches, such as molecular modeling, have accelerated the identification and optimization of these engineered cyclotides. Virtual docking and molecular dynamics studies [8,12] have guided the selection of scaffolds and graft positions, predicted membrane interactions, and even suggested cyclotides capable of binding virulence factors, such as bacterial neuraminidases, thereby expanding their action beyond direct membrane lysis to anti-virulence strategies.

Recent bioengineering approaches have not only focused on improving bacterial selectivity but have also, through ongoing rational design, addressed safety concerns, such as minimizing toxicity toward mammalian cells and hemolytic activity. Structure–activity studies have shown that modifying surface-exposed cationic residues can reduce hemolysis without sacrificing antibacterial potency [27,42].

Rational sequence modifications, including alterations in hydrophobic residues, charge distribution, and amphipathic balance, have been shown to reduce erythrocyte membrane disruption while preserving antibacterial activity [30,43,46]. This balance between efficacy and toxicity remains central to translation. Recent work on chimeric cyclotides demonstrated that grafting structural features from the Möbius and trypsin inhibitor subfamilies can modulate membrane interactions and bioactivity. In particular, the study highlighted the importance of preserving the CCK framework while altering surface-exposed residues associated with toxicity [46]. Another relevant observation was that the structural plasticity within cyclotide scaffolds enables sequence modifications without disrupting the overall fold. Nevertheless, these findings highlight the scaffold’s unique advantage: fine-tuning function through targeted mutations while retaining the stable cyclic cystine knot fold.

Computational approaches, including molecular dynamics simulations and machine learning-assisted peptide optimization, are increasingly used to predict hemolytic potential during early-stage design, thereby enabling prioritization of variants with improved selectivity profiles [45,47,48,49].

In addition, targeted delivery systems, lipidation strategies, and conjugation approaches may help reduce off-target membrane interactions and improve therapeutic indices in vivo [19,49]. Nevertheless, despite promising advances, comprehensive toxicity characterization remains limited for many cyclotides, and further in vivo studies are required to establish safe therapeutic windows and clarify long-term biocompatibility.

The structural complexity of cyclotides, particularly their head-to-tail cyclized backbone and multiple disulfide bonds, also presents important manufacturing challenges for large-scale therapeutic development. Nonetheless, to overcome these limitations, substantial progress has been made in recombinant and cell-free expression systems, including plant, bacterial, and yeast platforms, offering scalable, potentially cost-effective routes for cyclotide production. In-cell expression further enables the generation of large genetically encoded cyclotide libraries; together with chemical synthesis, these strategies have significantly expanded the feasibility of producing engineered cyclotides for pharmaceutical applications, thereby allowing rapid screening of variants that modulate specific molecular targets [14].

Cyclotides are ribosomally synthesized in plants [13]. Regarding synthesis strategies, chemical synthesis via solid-phase peptide synthesis (SPPS) remains one of the most widely used approaches for generating native and engineered analogs, offering several important advantages, such as enabling precise sequence control and chemical modification, incorporation of non-natural amino acids, isotope labeling, and rational structural modifications, facilitating and making it valuable for structure–activity studies and rational design. While precise, traditional SPPS-based production remains costly and labor-intensive for large-scale production of disulfide-rich macrocyclic scaffolds [13].

Most cyclotides are synthesized using Boc and Fmoc-based SPPS, followed by backbone cyclization and oxidative folding to achieve the native CCK topology [13,50]. Typically, the peptide chain is assembled on a highly acid-labile resin to obtain a fully side-chain-protected linear precursor upon mild cleavage. Backbone cyclization is then commonly performed in solution using coupling reagents such as HATU/DIPEA, followed by global side-chain deprotection, oxidative folding, and purification steps to obtain the final folded cyclotide [13,51]. However, oxidative folding of engineered cyclotides can be substantially impaired when inserted epitopes disrupt the native CCK topology, often resulting in low yields and reduced synthetic efficiency [13,44,51].

To address these limitations, recent studies have proposed modular plug-and-play synthetic strategies that allow bioactive epitopes to be introduced onto pre-folded cyclotide scaffolds [51]. This approach reduces two major bottlenecks associated with conventional cyclotide synthesis: the inefficient stepwise assembly of long grafted sequences and the impaired oxidative folding frequently observed after epitope insertion. The modular strategy also enables the incorporation of structurally complex epitopes, including sequences containing additional disulfide bonds or non-standard amino acids, thereby facilitating rapid diversification of cyclotide scaffolds for drug discovery applications [51]. Advances of biomimetic oxidative folding in organic solvents have also enabled the rapid formation of correctly folded cysteine-rich peptides with minimal accumulation of misfolded or dead-end intermediates. This approach substantially accelerates disulfide exchange reactions and improves folding efficiency, making it particularly advantageous for synthesizing structurally complex cyclotides with cystine-knot frameworks [52].

Despite these advances, some technical challenges remain. One important limitation is the potential epimerization during amide bond formation, particularly during final cyclization reactions involving unprotected amino acids. Although recent studies have reported minimal evidence of epimerization in selected examples, this effect may be sequence-dependent and may require careful optimization. The use of alternative coupling reagents, such as DIC/Oxyma, and the incorporation of C-terminal glycine residues have been suggested as strategies to minimize this issue [51].

Beyond purely chemical approaches, recent reviews highlight significant advances in recombinant expression systems in plants, bacteria, and yeast, as well as cell-free expression platforms, which are beginning to overcome yield limitations and reduce costs [14,53,54]. Techniques such as enzymatic ligation, native chemical ligation, and cell-free expression have increased yields and reduced costs, making the scalable manufacture of engineered cyclotides more viable [7]. Recombinant expression additionally enables the generation of genetically encoded cyclotide libraries for high-throughput screening against specific molecular targets [14]. Enzymatic ligation technologies and intein-mediated cyclization strategies have further improved production efficiency and broadened access to engineered cyclotides with pharmaceutical potential.

Together, these advances demonstrate that cyclotide production is progressively transitioning from small-scale experimental synthesis toward more scalable and modular manufacturing platforms. Nevertheless, improving folding efficiency, reducing production costs, and achieving robust large-scale manufacturing remain essential translational challenges for the future clinical development of cyclotide-based antibacterial therapeutics.

Beyond safety and production, pharmacokinetics and scalability remain major translational hurdles for cyclotides. Although their exceptional stability offers clear advantages, data on absorption, distribution, metabolism, and excretion remain limited. For example, pharmacokinetic studies on kalata B1 have shown poor oral bioavailability and rapid clearance, underscoring the need for strategies to improve systemic exposure and tissue distribution [55]. Recent engineering approaches have demonstrated promising solutions: lipidation of a cyclotide-based CXCR4 antagonist significantly enhanced half-life and in vivo activity [50], while grafting strategies combined with chemical plug-and-play synthesis approaches allow fine-tuning of pharmacological properties [51]. Still, addressing both pharmacokinetic optimization and cost-effective manufacturing will be essential to unlock the therapeutic promise of cyclotides in antibacterial drug development.

## 5. AI Opportunities and Future Perspectives

Looking ahead, integrating AI and machine learning (ML) into cyclotide research promises to accelerate the translation of natural peptide discovery into clinically viable antibacterial agents. Recent advances highlight the power of AI to complement classical screening and design strategies by rapidly analyzing and learning from vast databases of antimicrobial peptide sequences and structures, enabling the prediction of antimicrobial activity, toxicity, membrane affinity, and structural stability with far greater speed and accuracy than traditional methods. Additionally, ML algorithms can identify patterns in cyclotide sequences associated with high efficacy or low hemolysis, thereby guiding the rational design of optimized analogs [45,47,56].

The utility of AI and ML can be further amplified when coupled with omics-driven discovery pipelines. For instance, transcriptome sequencing has already been used to uncover novel cyclotides from *Viola arcuata*, illustrating how large-scale transcriptomic datasets can serve as training material for predictive models. Integrating such datasets with AI may support cyclotide discovery and facilitate the prioritization of candidates predicted to display favorable antibacterial, toxicity, and pharmacokinetic profiles [53,57]. Although computational approaches have already contributed to the identification and optimization of bioactive cyclotides and antimicrobial peptide analogs, including candidates that subsequently demonstrated experimental activity in vitro and, in some cases, in vivo (e.g., MCo-PG2) [30,45], most AI-driven predictions remain at an early stage and require rigorous experimental validation (MIC, MBC, hemolysis, toxicity, and in vivo efficacy studies) before conclusions regarding therapeutic performance can be drawn.

For instance, deep learning models trained on peptide–lipid interaction datasets have demonstrated the ability to identify sequence patterns associated with membrane interaction and reduced hemolytic potential, providing a complementary tool for rational cyclotide engineering [54]. Generative models and large-scale protein language models have emerged as exploratory tools for proposing novel cyclotide variants and grafted chimeras with predicted activity against specific MDR pathogens, but most generated candidates remain computational predictions, and only a limited number have undergone experimental validation. [55]. Coupled with molecular dynamics simulations and docking [8,12], AI may assist in prioritizing cyclotide candidates predicted to interact with bacterial membranes, virulence-associated targets, or biofilm-related proteins. Nevertheless, the application of AI to cyclotide discovery still faces limitations related to dataset size, structural diversity, and the scarcity of experimentally validated training datasets [58,59].

Moreover, besides AI tools helping to predict activity, they are contributing to the optimization of pharmacokinetic properties by predicting peptide stability, which is a critical step for selecting candidates suitable for systemic administration [33]. ML models can accurately predict peptide stability in gastrointestinal fluids based on sequence and physicochemical features, enabling digital screening for oral bioavailability and systemic suitability [60].

Furthermore, AI-driven prediction of membrane permeability and peptide uptake efficiency helps address delivery challenges, especially when combined with high-throughput synthesis and rapid in vitro validation, thereby accelerating the transition from scaffold selection to preclinical testing [61]. When combined with high-throughput synthesis and rapid in vitro validation, this data-driven approach can reduce the timeline from scaffold selection to preclinical testing. Altogether, these AI-enhanced strategies illustrate a paradigm shift: moving from empirical peptide discovery to rational, data-driven engineering of cyclotides as next-generation antibacterial therapeutics. However, even if these methods may accelerate candidate generation and prioritization, their translational impact remains dependent on experimental validation, larger datasets, and integration with iterative biological testing before clinical relevance can be established. Even though they offer opportunities to explore further the antimicrobial potential of cyclotides, such computational observations should be interpreted as hypothesis-generating, and supporting their future development as therapeutic candidates into clinically viable drugs, that must be supported by experimental evidence to address the growing threat of multidrug-resistant infections.

## 6. Conclusions

Cyclotides are a structurally distinct and functionally versatile class of plant-derived cyclic peptides that have demonstrated significant promise as templates for developing novel antibacterial agents. Their remarkable stability, stemming from the cyclic cystine knot motif, allows for extensive sequence variability while preserving conformational rigidity, an ideal property for engineering therapeutics with enhanced activity, selectivity, and pharmacokinetic profiles. The capacity of cyclotides to disrupt bacterial membranes, coupled with their emerging anti-virulence and antibiofilm activities, distinguishes them from conventional antibiotics and broadens their therapeutic relevance in the fight against MDR pathogens.

Despite these advantages, translational challenges persist, particularly in optimizing selectivity to reduce host cytotoxicity, improving oral bioavailability, and achieving scalable production. However, recent advances in peptide synthesis, recombinant expression, and computational design, particularly those supported by AI and ML, are contributing to overcoming some of these limitations. Notably, the ability to rationally design and fine-tune cyclotides through grafting or surface-modification strategies enables the generation of analogs with improved potency and selectivity profiles.

The convergence of bioinformatics, omics-based discovery, and in silico screening represents a paradigm shift, supporting the progression from natural cyclotide discovery toward more rational preclinical development pipelines. As synthetic biology and structure-based design continue to evolve, cyclotides are emerging as not only antimicrobial agents but also adaptable scaffolds for multifunctional peptide drugs. Their integration into next-generation therapeutic pipelines may help expand the repertoire of antimicrobial candidates and support efforts to address the ongoing challenge of MDR infections.

## Figures and Tables

**Table 1:** Antibacterial Activity and Hemolytic Properties of Plant-Derived Cyclotides.

Cyclotide	Family	Plant Source	Bacterial Species	Strain	MIC (μM)	MBC (μM)	HD50 (μM)	Ref.
Cliotide T1	Fabaceae	*Clitoria ternatea*	*Escherichia coli*	ATCC 700926	1.1	NR	7.1	[32]
			*Pseudomonas aeruginosa*	ATCC 39018	4.7			
			*Klebsiella pneumoniae*	ATCC 13883	2.7			
Cliotide T4			*Escherichia coli*	ATCC 700926	1.0	NR	8.4	
			*Pseudomonas aeruginosa*	ATCC 39018	7.5			
			*Klebsiella pneumoniae*	ATCC 13883	5.5			
Cliotide T7 (Cter R)			*Escherichia coli*	ATCC 700926/25922	3.1–40	NR	>100	[29]
			*Pseudomonas aeruginosa*	ATCC 27853	0.62			
Cliotide T10 (Cter B)			*Escherichia coli*	ATCC 25922	>100	NR	NR	[28,32]
			*Pseudomonas aeruginosa*	ATCC 27853	20			
Cliotide T15			*Escherichia coli*	ATCC700926	0.5–2.5	NR	NR	[29]
Cliotide T16					2.4			
Cliotide T19					0.6			
Cliotide T20					0.5–10			
Cter E			*Pseudomonas aeruginosa*	ATCC 27853	>100	NR	NR	[28]
Cter G					0.62			
			*Escherichia coli*	ATCC 25922	40	NR	NR	
Panitide L2	Poaceae	*Panicum laxum*		NR	2.5	NR	NR	[33]
Kalata B1	Rubiaceae	*Oldenlandia affinis*		ATCC 25922	4	~6	~12	[23,27,34,35]
			*Pseudomonas aeruginosa*	ATCC 27853	32	>64		
			*Klebsiella oxytoca*	NR	~55	NR		
Kalata B2 *			*Staphylococcus aureus*	ATCC 25923	50	NR	NR	[28,36]
			*Pseudomonas aeruginosa*	ATCC 27853	>100			
					1.25			
Kalata B7			*Escherichia coli*	ATCC 25922	>100	NR	NR	[28]
			*Pseudomonas aeruginosa*	ATCC 27853	20			
Kalata B13					80	NR	NR	
Varv A/Kalata S *			*Staphylococcus aureus*	ATCC 25923	>100	NR	NR	[23,28]
			*Escherichia coli*	ATCC 25922	>100			
			*Proteus vulgaris*	NR	54–>100			
Circulin A		*Chassalia parviflora*	*Staphylococcus aureus*	NR	0.19>100	NR	NR	[23]
			*Pseudomonas aeruginosa*	NR	25–48			
Circulin B			*Proteus vulgaris*	NR	6–>100	NR	NR	[22]
			*Klebsiella oxytoca*	NR	8–16	NR		
			*Staphylococcus aureus*	NR	13–>100	NR		
			*Escherichia coli*	NR	0.41–>100	NR		
			*Escherichia coli*	ATCC 25922	6.4	NR	11.6	[37]
Chassatide C7		*Chassalia chartacea*			6.6	NR	25.5	
Chassatide C8			*Pseudomonas aeruginosa*	NR	13.5	NR	NR	[38]
Cyclopsychotride A		*Psychotria longipes*	*Proteus vulgaris*	NR	13.2	NR	NR	[38]
			*Klebsiella oxytoca*	NR	5.8	NR		
			*Staphylococcus aureus*	NR	39	NR		
			*Micrococcus luteus*	NR	48	NR		
			*Escherichia coli*	NR	1.55	NR		
			*Streptococcus salivarius*	ATCC 13419	5.9	NR		
Hedyotide B1		*Hedyotis biflora*	*Escherichia coli*	ATCC 25922	3.4	NR	NR	[32,39]
					1.5	NR		
Hedyotide B10			*Streptococcus salivarius*	ATCC 13419	2.1	NR	NR	[39]
			*Escherichia coli*	ATCC 25922	1.8	NR		
Hedyotide B11			*Streptococcus salivarius*	ATCC 13419	2.2	NR	NR	
Gere 1		*Geophila repens*	*Escherichia coli*	ATCC 25922	4	NR	NR	[31]
			*Pseudomonas aeruginosa*	ATCC 27853	7.9	NR	NR	
Cycloviolacin O2	Violaceae	*Viola odorata*	*Staphylococcus aureus*	ATCC 29213	10	NR	4–6	[27,28,36]
			*Acinetobacter baumannii*	ATCC 19606	4.2	NR		
			*Bacillus subtilis*	CMCC(B)63501	2.1	NR		
			*Pseudomonas aeruginosa*	ATCC 27853	10	NR		
			*Staphylococcus aureus*	ATCC 29213	20	NR		
Cycloviolacin O3			*Escherichia coli*	ATCC 25922	10	NR	NR	[28]
			*Pseudomonas aeruginosa*	ATCC 27853	10			
			*Staphylococcus aureus*	ATCC 29213	10			
Cycloviolacin O19			*Escherichia coli*	ATCC 25922	10	NR	NR	
			*Pseudomonas aeruginosa*	ATCC 27853	10			
			*Staphylococcus aureus*	ATCC 29213	>100			
Tricyclon A		*Viola tricolor*	*Escherichia coli*	ATCC 25922	>100	NR	NR	

Abbreviations: MIC, minimum inhibitory concentration; MBC, minimum bactericidal concentration; HD50, concentration causing 50% hemolysis of human erythrocytes; NR, not reported; * Also present in Violaceae.

## Data Availability

No new data were created or analyzed in this study. Data sharing is not applicable to this article.

## Abbreviations

The following abbreviations are used in this manuscript:

MDR	Multidrug-resistant
AMPs	Antimicrobial peptides
CCK	Cysteine cyclic knot
AI	Artificial intelligence
CyO2	Cycloviolacin O2
PE	Phosphatidylethanolamine
MIC	Minimum inhibitory concentration
ML	Machine learning
